# Supplementary material for: The interactome of intact mitochondria by cross-linking mass spectrometry provides evidence for coexisting respiratory supercomplexes
Source: Mol Cell Proteomics. 2017 Dec 8;17(2):216–32. doi: 10.1074/mcp.RA117.000470 (PMC5795388; doi:10.1074/mcp.RA117.000470)
Supplement: Supplemental Data [file supp_RA117.000470_133922_0_supp_23978_fzffwf.zip › spectra_annotation/mito_DR_spectra_annotation/9-1-9-1-4-1.pdf]

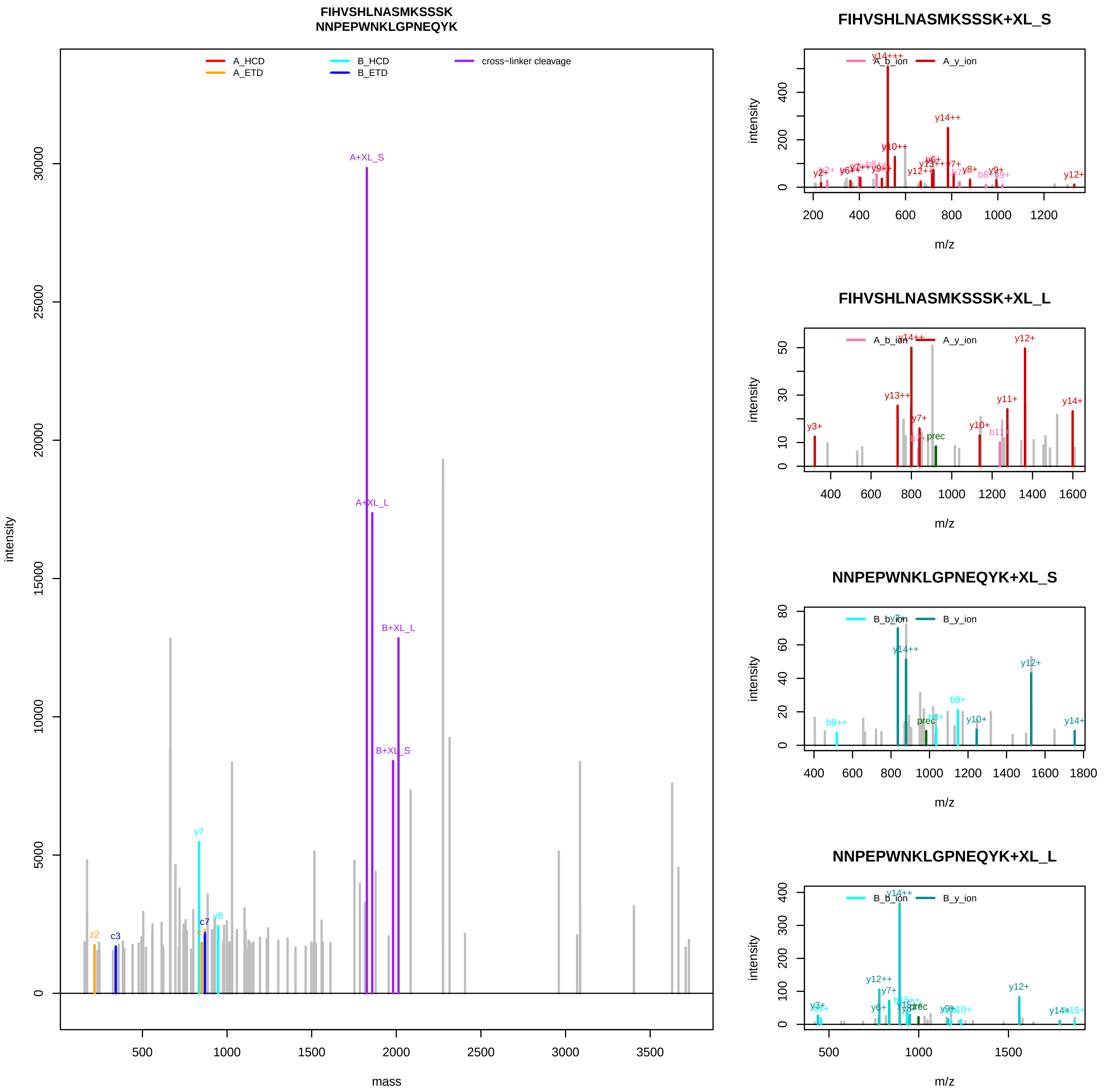
FIHVSHLNASMKSSSK
NNPEPWNKLGPNEQYK
A_HCD
A_ETD
B_HCD
B_ETD
cross−linker cleavage
A+XL_S
A+XL_L
B+XL_L
B+XL_S
y7
y8
c7
c3
z2
intensity
mass
0
5000
10000
15000
20000
25000
30000
500
1000
1500
2000
2500
3000
3500
FIHVSHLNASMKSSSK+XL_S
A_b_ion
A_y_ion
y14+++
y14++
y10++
y12+
m/z
FIHVSHLNASMKSSSK+XL_L
y14++
y12+
y13++
y11+
y14+
y3+
y7+
y10+
b11+
prec
NNPEPWNKLGPNEQYK+XL_S
B_b_ion
B_y_ion
y14++
y12+
b9+
b8++
y10+
y14+
prec
NNPEPWNKLGPNEQYK+XL_L
y14++
y12++
y12+
prec
